# Dicentric chromosome assay using a deep learning-based automated system

**DOI:** 10.1038/s41598-022-25856-1

**Published:** 2022-12-21

**Authors:** Soo Kyung Jeong, Su Jung Oh, Song-Hyun Kim, Seungsoo Jang, Yeong-Rok Kang, HyoJin Kim, Yong Uk Kye, Seong Hun Lee, Chang Geun Lee, Moon-Taek Park, Joong Sun Kim, Min Ho Jeong, Wol Soon Jo

**Affiliations:** 1 Research Center, Dongnam Institute of Radiological & Medical Sciences (DIRAMS), 40 Jwadong-Gil, Jangan-Eup, Gijang-Gun, Busan, 46033 Republic of Korea; 2 Department of Microbiology, Dong-A University College of Medicine, Daeshingongwon-Gil 32, Seo-Gu, Busan, 602-714 Republic of Korea; 3 SierraBASE Co. Ltd., 77 Cheongam-Ro, Nam-Gu, Pohang, 37673 Republic of Korea; 4 Division of Advanced Nuclear Engineering, POSTECH, 77 Cheongam-Ro, Nam-Gu, Pohang, 37673 Republic of Korea; 5 College of Veterinary Medicine and BK21 Plus Project Team, Chonnam National University, 77 Yongbong-Ro, Buk-Gu, Gwangju, 61186 Republic of Korea

**Keywords:** Chromosomes, Cytogenetics

## Abstract

The dicentric chromosome assay is the “gold standard” in biodosimetry for estimating radiation exposure. However, its large-scale deployment is limited owing to its time-consuming nature and requirement for expert reviewers. Therefore, a recently developed automated system was evaluated for the dicentric chromosome assay. A previously constructed deep learning-based automatic dose-estimation system (DLADES) was used to construct dose curves and calculate estimated doses. Blood samples from two donors were exposed to cobalt-60 gamma rays (0–4 Gy, 0.8 Gy/min). The DLADES efficiently identified monocentric and dicentric chromosomes but showed impaired recognition of complete cells with 46 chromosomes. We estimated the chromosome number of each “Accepted” sample in the DLADES and sorted similar-quality images by removing outliers using the 1.5IQR method. Eleven of the 12 data points followed Poisson distribution. Blind samples were prepared for each dose to verify the accuracy of the estimated dose generated by the curve. The estimated dose was calculated using Merkle’s method. The actual dose for each sample was within the 95% confidence limits of the estimated dose. Sorting similar-quality images using chromosome numbers is crucial for the automated dicentric chromosome assay. We successfully constructed a dose–response curve and determined the estimated dose using the DLADES.

## Introduction

In cases of inadvertent radiation exposure, such as large-scale radiation catastrophes, terrorism, and occupational radiation accidents, quantifying the radiation absorbed is a prerequisite for treatment-related decisions^1^. In the absence of physical dosimetry, biological dosimetry is critical in assessing the exposure dose. Several biodosimetry methods have been investigated, including physiological^2,3^ and biological markers^4–7^. Chromosomal aberration is a radiation-specific biomarker, and scoring such aberrations is widely regarded as an effective technique for identifying radiation effects. Radiation-induced DNA double-strand breaks activate cellular damage repair mechanisms. However, misrepair of chromosomes results in chromosomal aberrations, typically dicentric chromosomes and translocations^8–11^. Dicentric chromosomes are unstable chromosomal aberrations that eventually induce cell death; therefore, they have a low background level in humans^12,13^ and increase dose-dependently with radiation exposure^14–16^. The dicentric chromosome assay is the gold-standard method, which is well established and internationally standardized^17^.

Despite its dose-estimation accuracy, the dicentric chromosome assay is burdensome for implementation in large-scale radiation accidents owing to its time-consuming approach and the need for expert scorers to identify chromosomal abnormalities^17,18^. For a reasonably accurate dose estimate, the International Atomic Energy Agency (IAEA) and International Organization for Standardization (ISO) guidelines recommend scoring 500 cells or 100 dicentrics. Especially at low doses, it requires 1–2 days to obtain results because for the analysis, 500 complete metaphases with 46 chromosomes must be selected, and two expert scorers intercompare to obtain reliable results. On the contrary, studies have shown that analyzing the number of dicentrics in 50 cells is sufficient for a triage in mass casualty situations^19,20^. However, a decreased time dependent on the number of cells to be analyzed results in a decrease in accuracy^20,21^.

Chromosome-identifying automatic image analysis systems have been recently investigated to overcome this challenge^22–25^. These systems offer the advantage of rapid large-scale image analysis. However, manual and automatic dicentric chromosome scoring approaches differ, and the constraints of automated programs limit fully automatic outcomes using manual scoring statistics. For instance, manually scoring dose–response curves involves screening entire cells with 46 centromeres and validating that the frequency of dicentric chromosomes follows a Poisson distribution under low-LET acute whole-body exposure conditions^17,26^. After fitting the dose–response curve, the frequency of dicentric chromosomes is estimated. However, automated scoring data show that the number of dicentric chromosomes increases radiation dose-dependently but does not always follow the Poisson distribution. Several trials have been conducted to facilitate dose–response curve generation, such as using different models^27^ or an expert review process for selecting whole cells with 46 chromosomes^28^. Furthermore, the final result is more accurate because experts confirm the dicentric chromosome candidates^21^. However, the human review process is time-consuming and restricts large-scale sample processing. Recently, Endesfelder et al.^29^ proposed a method to limit the number of chromosomes to ensure that dicentric chromosomes follow a Poisson distribution and a dose–response curve can be constructed without human evaluation. Previously, we constructed a deep-learning-based automatic dose-estimation system (DLADES) that can distinguish dicentric and monocentric chromosomes^30^. In this study, we proposed and evaluated the application of the DLADES for constructing a dose–response curve using manual scoring statistics and estimating the dose without human assessment.

## Results

### Analysis of chromosome distribution

**Figure 1:**
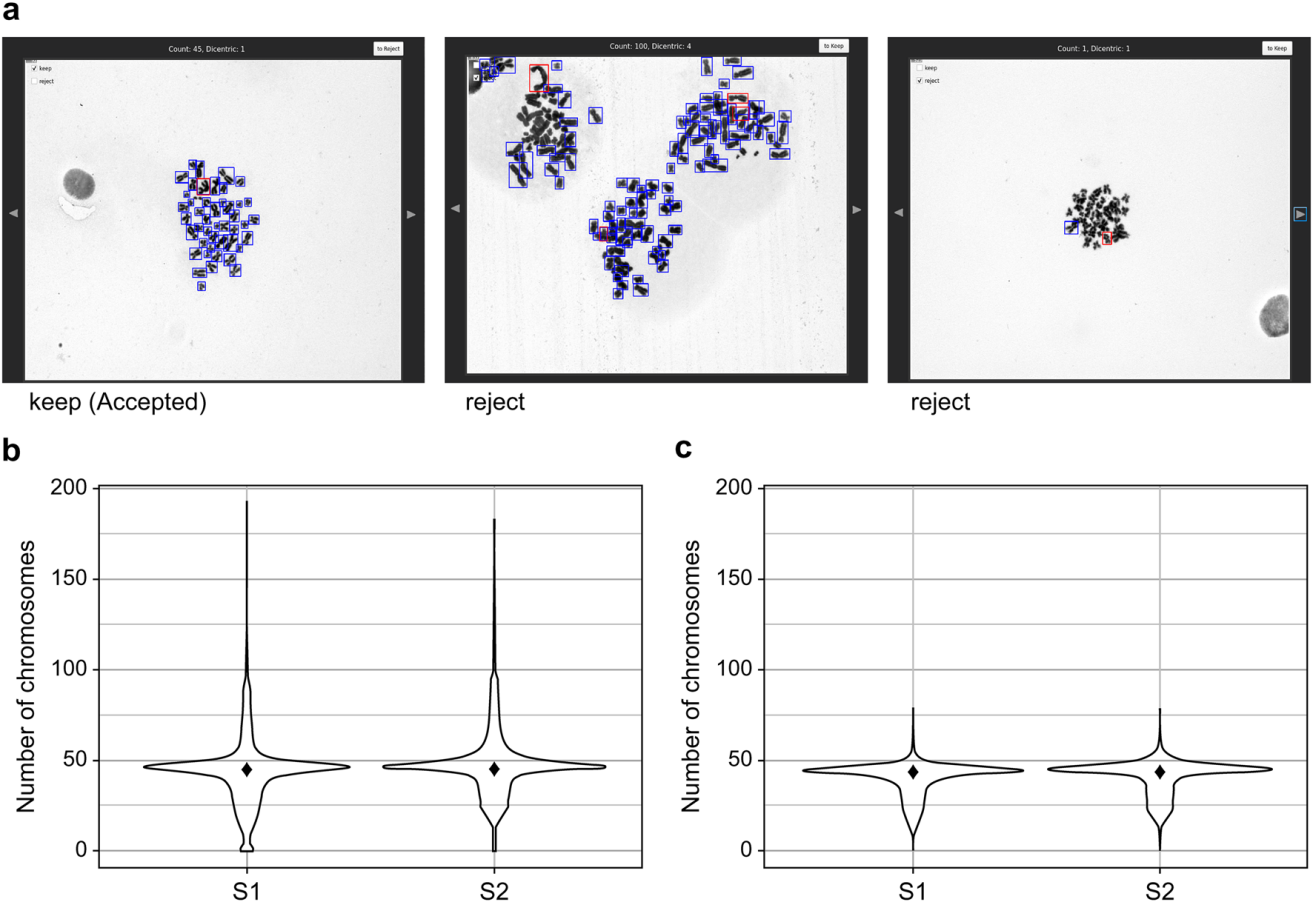
Distribution of chromosome numbers identified by the automatic system. (**A**) DLADES-detected monocentric and dicentric chromosomes are denoted using blue and red frames, respectively. The upper left corner of the program window indicates whether the image is “Accepted.” (**B**) Automatically scored chromosomes from the total data of two individuals. (**C**) Automatically scored chromosomes from the “Accepted” data of two individuals. ◆: Median.

We irradiated blood samples from two healthy participants in their 30 s (S1 female, S2 male) at 0, 0.5, 1, 2, 3, and 4 Gy (dose rate 0.8 Gy/min) to construct a dose–response curve. A single-cell image was captured by spreading loosely dispersed cells in a fixed solution over a slide. The scanning sensitivity of the Metafer4 System was set to 7 to capture cells in the metaphase, ensuring consistent identification of metaphase cells across all samples. Next, the DLADES was used to detect monocentric and dicentric chromosomes after converting the images to JPEG format without human involvement (Fig. 1a). S1 and S2 had a mean of 44.04 and 47.88 chromosomes, respectively, with distributions ranging from 0 to more than 100 (Fig. 1b and Table 1). If the number of chromosomes exceeded 100, more than two cells were captured in the image (Fig. 1a, second panel). In contrast, if 10 or fewer chromosomes were identified, the cell was either not in the metaphase or the chromosome quality was poor (Fig. 1a, third panel). Particularly, the dicentric frequency was affected if the DLADES misidentified a cell structure or chromatin as a chromosome due to its poor shape. Our system automatically classified images as “Accepted” or “Filtered” to obtain more accurate results during the identification process^30^. We initially examined the chromosome distribution, which was classified as “Accepted.” As depicted in Fig. 1c and Table 1, both mean number of “Accepted” chromosomes (S1 = 41.12, S2 = 42.10) and the standard deviation (S1 = 9.75, S2 = 9.75) were slightly lower than those in the total dataset. This implies that the “Accepted” step clustered chromosomes around the mean and standardized the chromosome image quality. In low-LET acute whole-body exposures^17^, the number of dicentric chromosomes follows the Poisson distribution. We confirmed that most of the “Accepted” data followed this distribution, except two (S1-3 Gy and S1-4 Gy) out of 12 data points (Supplementary Table S1). However, most of the total data were overdispersed, emphasizing the importance of using harmonized images when analyzing the frequency of dicentric chromosomes using an automation program without human review.

**Table 1:** Summary of automatically identified chromosome numbers from the Total and Accepted data.

		Min	Mean	Median	Max	SD
Total	S1	0	44.04	45	192	18.54
	S2	0	47.88	47	183	17.63
Accepted	S1	1	41.12	45	81	9.75
	S2	0	42.10	46	81	9.75

Min: minimum, Max: maximum, SD: standard deviation.

### Overdispersion of dicentric chromosomes by chromosome number

**Figure 2:**
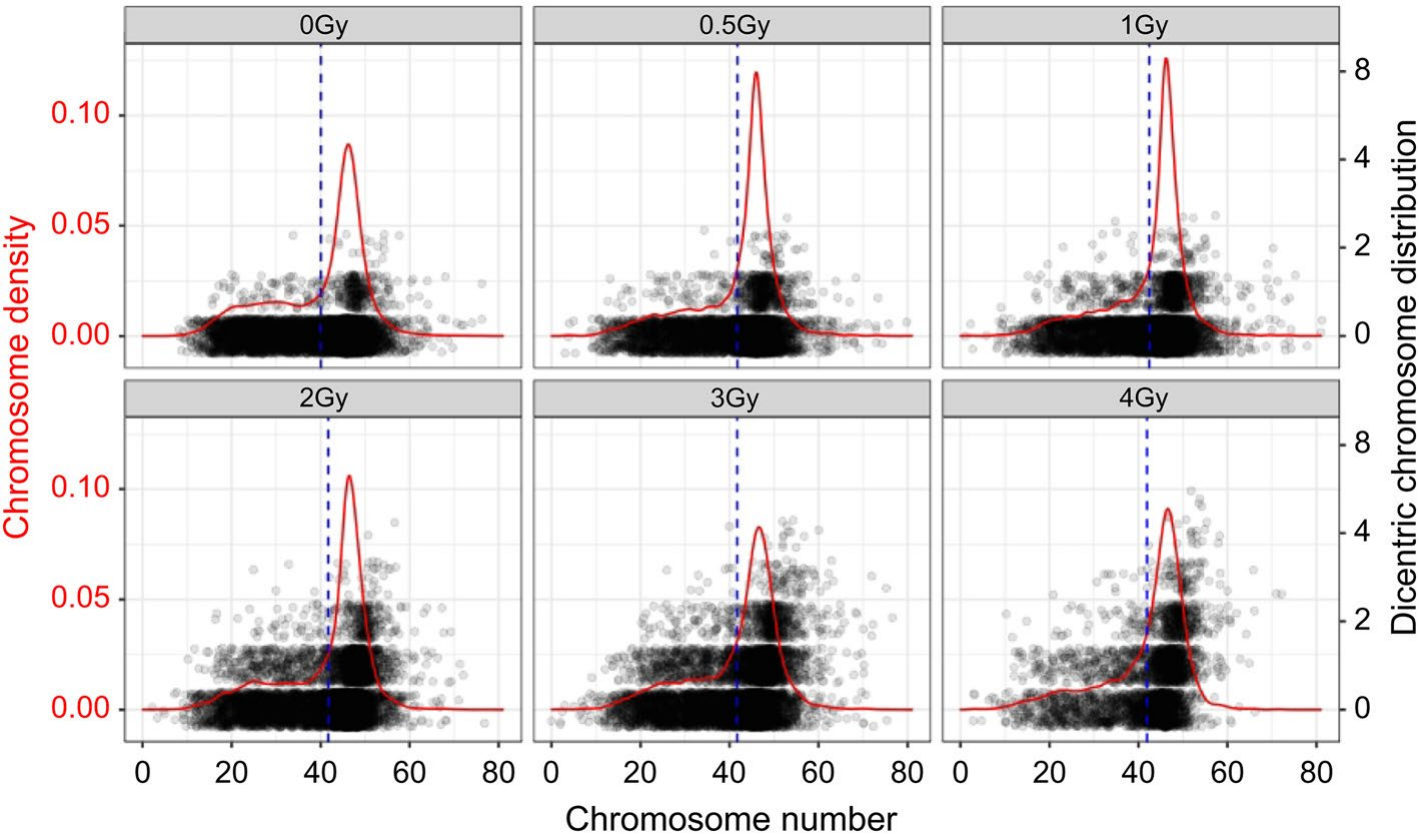
Dicentric chromosome distribution and chromosome density by dose (Gy) in “Accepted” data. The “Accepted” data obtained from two donors is pooled by dose. The density plot represents the chromosomal density (solid red line); the jitter points plot indicates the distribution of the dicentric chromosome. The blue dashed line denotes the mean.

The dicentric chromosomes mostly followed the Poisson distribution in the “Accepted” image, but when the datasets were pooled, overdispersion was revealed at 3 and 4 Gy (Supplementary Table S2). Recently, Endesfelder et al^29^. found that metaphase chromosomal number influences the overdispersion of dicentric chromosomes. To determine if chromosome number affects overdispersion, we examined the frequency of dicentric chromosomes according to chromosome number. In the pooled data, the average number of chromosomes for each dose was 40.03–42.42, with a median value of 45–46. For each dose, the median values were concentrated (Fig. 2 and Table 2). However, outlier data points were observed on dicentric chromosomes. Certain dicentric chromosome data points were far from the median value, which could have altered the dicentric distribution. Using within 1.5 × interquartile range (IQR) method, which ensures better harmonization and minimizes data loss, we investigated whether the number of chromosomes affected the dicentric chromosome overdispersion. As shown in Supplementary Table S3, the pooled data for 4 Gy of S1 followed a Poisson distribution. In the case of 3 Gy, S1 and S2 dispersion indices were closer to 1 at within 1.5 × IQR than the “Accepted” number; however, the 3 Gy points of S1 remained overdispersed, influencing the pooled result. In conventional scoring, a human scorer used a good-quality image to validate a complete cell with 46 chromosomes and locate dicentric chromosomes. This implies that the human scorer-based sorting enables image acquisition under the same conditions. The DLADES permits the sorting of good-quality images but not complete cells. Therefore, similar image conditions can be obtained by considering the chromosome number. We confirmed that only 3-Gy data showed pooled overdispersion. These findings validated the significance of an automated system to accurately identify monocentric and dicentric chromosomes and extract the frequency of the dicentric chromosome by selecting images of similar quality and condition.

**Table 2:** Summary of automatically identified chromosome numbers from Accepted pooled data.

Gy	Min	Mean	Median	Max	SD
0	8	40.03	45	76	10.53
0.5	3	41.77	45	80	9.45
1	1	42.42	46	81	9.01
2	2	41.70	46	77	9.83
3	0	41.72	45	77	9.91
4	3	41.94	45	72	9.51

Min: minimum, Max: maximum, SD: standard deviation.

### Constructing dose–response curves using automated scoring

**Figure 3:**
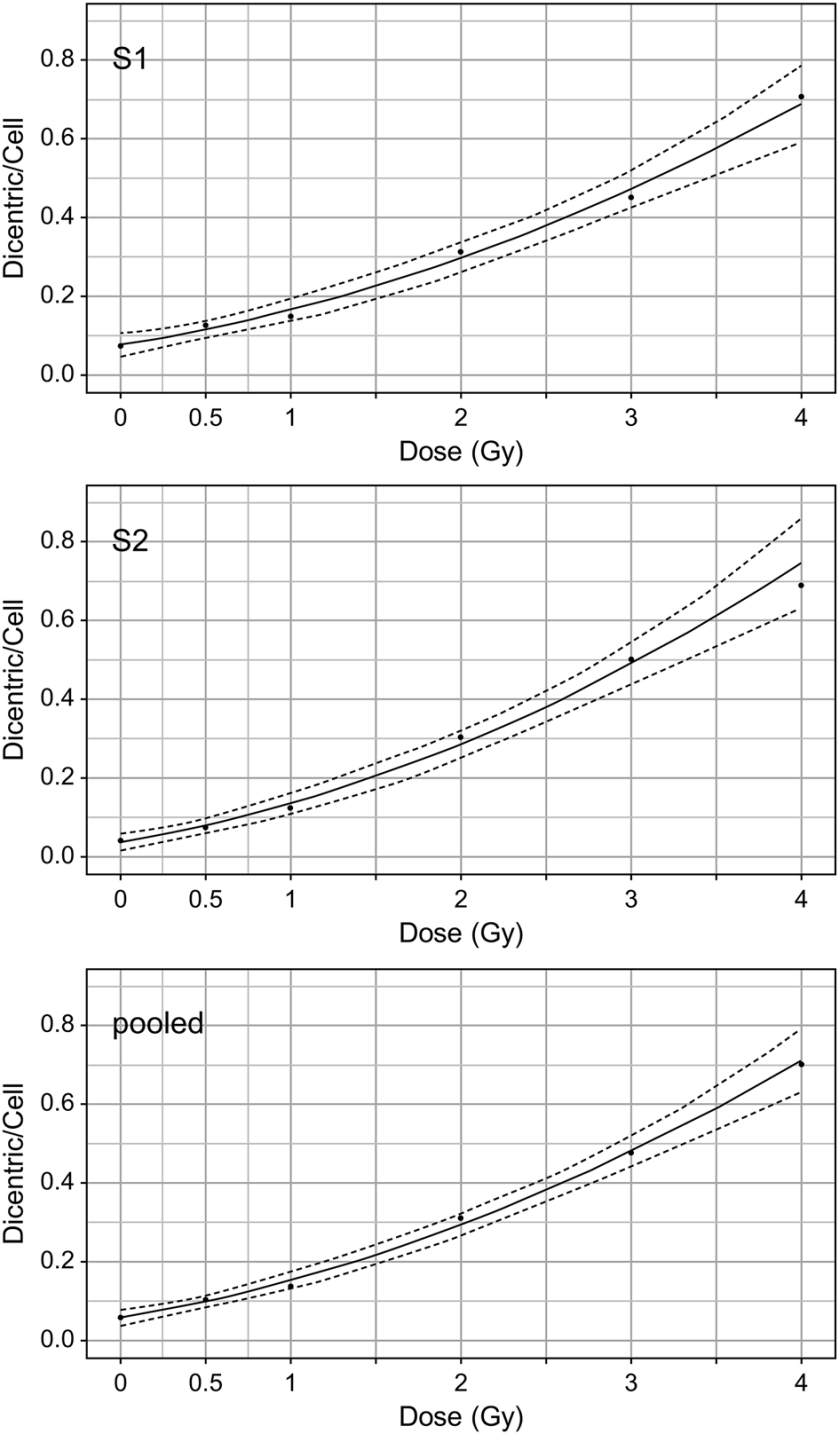
Dose–response curves were obtained from S1, S2, and pooled data. The curves were fitted using linear-quadratic Quasi-Poisson regression models (solid line) with 95% confidence intervals (dashed line). The yield at each dose was calculated after excluding the outliers of the chromosome number and expressed as a point.

**Figure 4:**
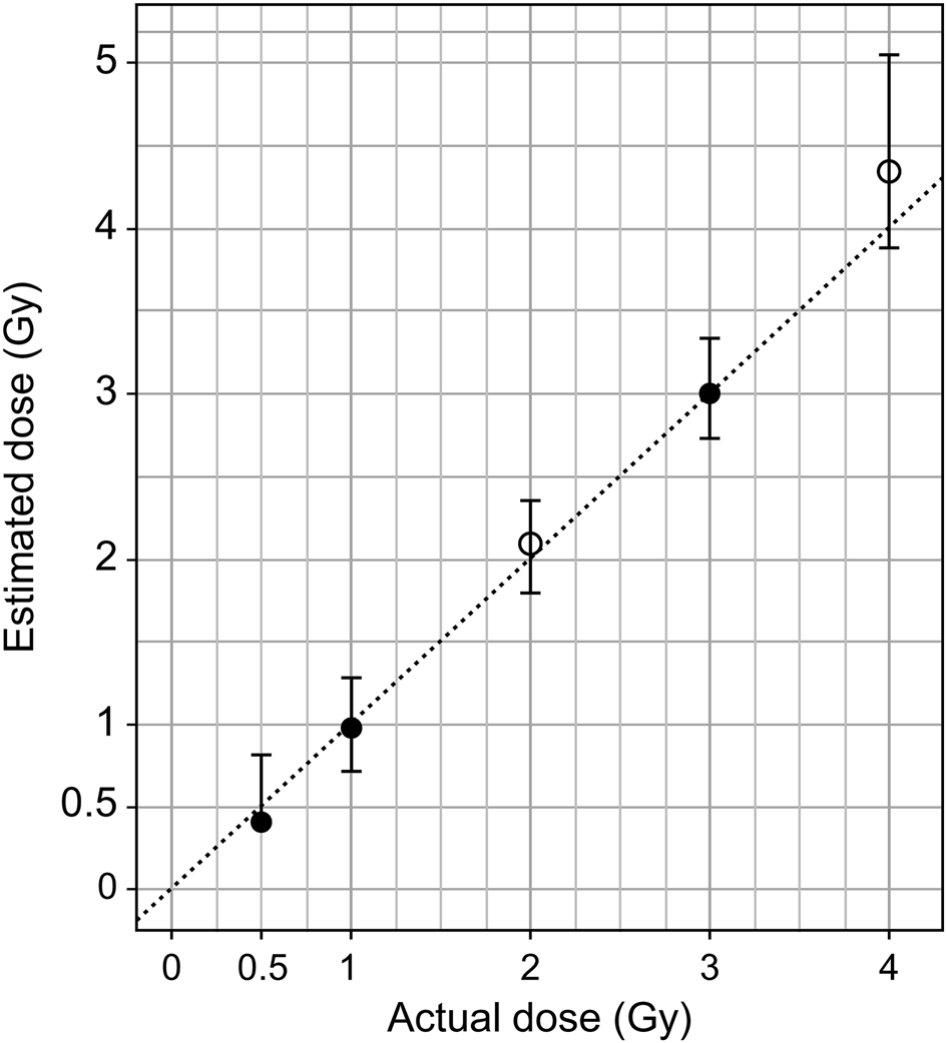
Estimated dose of the blind samples was obtained from two donors. The open and closed circles represent blind test samples 1 and 2, respectively. Merkle’s approach was used to calculate the estimated dose. For each dose, the upper and lower lines parallel to the *x*-axis represent the estimated doses of the upper and lower confidence limits, respectively.

We constructed a dose–response curve using R, as specified in IAEA EPR-Biodosimetry 2011^17^ because the dicentric chromosome assay dose–response curve conforms to a linear-quadratic model (yield = C + α × D + β × D^2^) for low-LET radiation. S1 and S2 data, as well as pooled data, were used to calculate the coefficients of the fitted curves (Fig. 3 and Table 3). Each of the three fitted curves had coefficients that rejected the null hypothesis at the 0.05 significance level. We used a pooled fitted curve to estimate the blind tests after conducting a *z*-test to compare the dose–response curves of the two donors^31^, but found no significant difference (a: *z* =  − 2.9, *p* = 0.61; b: *z* =  − 0.49, *p* = 0.69). For the blind test, metaphase cell images were obtained from irradiated blood samples of 29- and 39-year-old males. Identification of dicentric chromosomes using the DLADES revealed that all data points were overdispersed but only 0.5-Gy points remained overdispersed in the “Accepted” and outlier removed data (Supplementary Table S4). After adjusting the blind test data by excluding values outside the 1.5 IQR, we estimated the dicentric chromosome frequency for each dose (Table 4). Next, using a pooled fitted curve, we compared the estimated and actual doses (Fig. 4 and Table 4). The estimated dose was calculated according to Merkle’s approach based on the IAEA 2011 presentation^17,32^. The lower 95% confidence limits on the observed yield of the 0.5-Gy sample was marked NA because it did not intersect with the upper curve equation. The actual dose for the five blind samples was included in the 95% confidence limits of the estimated dose. In the 3-Gy blind sample, the difference between the estimated and actual doses was minimal (< 0.01). These findings confirmed that a dose–response curve could be constructed by applying conventional scoring statistical methods using the DLADES to calculate the estimated dose without review by human scorers.

**Table 3:** Calibration curve coefficients and standard errors for individuals S1 and S2, and the pooled data set.

Data set		Estimate	Std. error	t-test	*p*
S1	C	0.08	0.01	7.36	0.01
	α	0.07	0.02	3.34	0.04
	β	0.02	0.01	3.55	0.04
S2	C	0.04	0.01	5.28	0.01
	α	0.07	0.02	4.07	0.03
	β	0.03	0.01	4.35	0.02
Pooled	C	0.06	0.01	8.26	0.00
	α	0.07	0.01	5.02	0.02
	β	0.02	0.00	4.89	0.02

*C* is the constant of the calibration curve equation, and α and β indicate the coefficients of D and D^2^, respectively.

**Table 4:** Estimated dose of the blind test.

	Actual dose (Gy)	Estimated dose(Gy)	Estimated dose (Gy) 95% confidence limits	
			Upper	Lower
Blind test 1	0.5	0.40	0.81	NA
	1	0.98	1.27	0.71
	3	3.01	3.33	2.74
Blind test 2	2	2.08	2.35	1.81
	4	4.33	5.05	3.88

The estimated dose was calculated according to Merkle’s approach^17,30^.

## Discussion

The conventional dicentric chromosome assay is the most reliable approach for measuring the level of absorbed radiation in victims of radiation accidents among the various biological dose-assessment methods. Therefore, the entire process, from acquiring metaphase cells to scoring and identifying chromosomes, has been well-harmonized by IAEA 2011^17^ and ISO 2014^18^. However, the dicentric chromosome assay requires trained experts, is time-consuming, especially the scoring process, and has limited utility for processing a large number of samples. Various laboratories have collaborated to standardize the biological dosimetry method to overcome these challenges, validating its accuracy and performance^19,33–35^ and minimizing the scoring time^35–37^.

Recently, methods to quickly process many samples using a chromosome-recognition automatic system have also been studied^21,23,24,29^. For instance, Rogan's group has developed Automated Dicentric Chromosome Identifier and Dose Estimator (ADCI) software and is conducting active research^25,38–40^. We also investigated a method for automatically constructing dose curves and calculating the absorbed radiation dose using the DLADES, an automated dicentric chromosome scoring system based on a deep-learning technique^30^. The DLADES was trained with reliable data because three cytogenetics experts^33,41^ generated the training data stably using the bounding box method. Blood samples from two healthy donors in their 30 s were exposed to cobalt-60 gamma rays at a 0.8-Gy/min dose rate over six dose ranges from 0 to 4 Gy. Metaphase cells were induced following the IAEA 2011 protocol^17^, and images were captured using the Metafer4 System. The captured images were exported to JPEG format, and the monocentric and dicentric chromosomes for each dose were counted using the DLADES. The number of dicentric chromosomes increased dose-dependently. However, the results differed from those obtained using conventional dicentric chromosome assays. The dicentric chromosome follows a Poisson distribution in a conventional dicentric chromosome assay using low-LET radiation. As shown in Supplementary Table S1, most doses were overdispersed. Romm et al. attributed this outcome to the image quality^21^. In the conventional assay, the reviewer selects complete cells with 46 chromosomes, resulting in images of similar quality and condition.

However, the automatic system does not classify entire cells when analyzing the image. Therefore, the variable appearance of chromosomal types in captured images is a limitation of automatic scoring systems similar to manual scoring. We resolved this problem using the DLADES filtering function to sort the “Accepted” images. DLADES accurately counted and identified dicentric and monocentric chromosomes (97% and 90%, respectively)^30^. Therefore, we attempted to sort similar-quality images by “Accepted” classification of the DLADES, yielding an average number of chromosomes in S1 and S2 images of 41.12 and 42.10, respectively, with a standard deviation of 9.75 for both; thus, “Accepted” data were clustered around the mean compared to the total dataset. By excluding the 1.5 × IQR outlier, images with similar conditions were obtained, causing the mean and standard deviation to increase slightly from 42.99 to 43.35 and from 7.07 to 8.19, respectively.

By sorting similar condition images, our goal was to determine whether the dicentric chromosomes followed a Poisson distribution. We confirmed that most doses followed a Poisson distribution, except for 3 Gy of S1. In the case of S1 3 Gy, we tried to exclude up to 1 × outliers; however, this increased data loss and the remaining data did not follow the Poisson distribution. Poor sample conditions may explain the lower average number of chromosomes and a higher standard deviation in 3 Gy of S1 than in other samples. However, “Filtered” data represented 28.21% of the total data, which is lower than the average loss rate of 34.33%. Thus, in our future research, we will focus on the confounding factors affecting the data.

We successfully constructed a dose–response curve using the general linear model recommended by IAEA 2012 because our data, except for 3 Gy in S1, followed the Poisson distribution. The absorbed radiation dose should be quickly estimated to treat victims of a radiation accident. Therefore, the estimated dose should be categorized into four ranges (1–2, 2–4, 4–6, and > 6 Gy) for rapid estimation, and information for medical treatment should be provided^18^. We irradiated the blood with 0.5, 1, 2, 3, and 4 Gy doses and calculated the estimated dose to determine whether the dose–response curve constructed using the automatic system could accurately reflect clinical triage. In all the samples, the actual dose was within the 95% confidence intervals of the estimated dose, and the difference between the estimated and actual doses was minimal. Therefore, the medical treatments were successfully categorized. However, this study did not include the construction of a dose–response curve or estimating doses higher than 4 Gy. The limited metaphase cell yield at high doses challenged obtaining a sufficient number of samples. However, a method must be established for samples with exposures above 4 Gy to comply with the clinical triage system.

Furthermore, estimating doses at exposures below 1 Gy necessitates greater precision. According to Korea’s Enforcement Decree of the Nuclear Safety Act (Article 2 and attached Table 1)^42^, the dose limits for radiation workers are 50 mSv per year and 100 mSv in five consecutive years. When the dose limit is exceeded, the person is classified as a “person with an abnormal reading,” and safety measures should be taken. Therefore, to prepare for the risk of radiation exposure accidents, a method to estimate doses below 0.1 Gy is required in the radiation work environment. The dicentric chromosome assay identifies a low threshold dose of 0.1 Gy^43^ and requires scoring more than 5000 cells for statistically significant results^17^, which is difficult to achieve using conventional scoring methods. Therefore, an automatic scoring system can compute legal norms in the event of radiation workplace accidents. However, in this study, we did not evaluate whether it is possible to estimate the exposure at doses greater than 4 Gy and less than 0.5 Gy; further research is warranted to confirm these aspects.

In summary, we constructed an effective dose–response curve using an automated system and calculated the estimated dose for the unknown sample to validate the outcome. In this study, we confirmed that accurate and rapid dose assessments could be facilitated by obtaining results using an automatic system without human review.

## Materials and methods

### Sample preparation

The study was conducted in accordance with the tenets of the Declaration of Helsinki, and the protocol was approved by the Institutional Review Board of the Dongnam Institute of Radiological and Medical Sciences (DIRAMS) for all experimental procedures (Approval No. D-1602-002-001). Prior to inclusion in the study, informed consent was obtained from all participants under the supervision of the IRB of the DIRAMS via signing of a consent form containing the necessary details about the study. All methods were performed in accordance with the relevant guidelines and regulations. We enrolled healthy participants who had not been exposed to ionizing radiation for over 3 months. Peripheral blood samples were collected in lithium-heparin tubes (BD Biosciences, San Jose, CA, USA) and irradiated with Cobalt-60 gamma rays (Gamma Beam X-200; Best Theratronics Ltd., Ottawa, Canada) at a dose rate of 0.8 Gy/min. To obtain dependable results, we constructed an in vitro irradiation setting resembling the in vivo situation. Briefly, the blood sample was irradiated in a 37 °C water phantom, and calibration was performed using a farmer-type ion chamber (TM300013, PTW) for accuracy. The farmer-type ion chambers were calibrated according to IAEA Technical Report Series (TRS)-398 and Korean standards^44,45^, and the calibration factor was 0.05400 Gy/nC at a source to surface distance (SSD) of 100 cm and the blood sample was irradiated at an SSD of 77 cm to achieve 0.8 Gy/min. The uncertainties include statistical uncertainties and systemic uncertainties such as beam homogeneity, positional reproducibility, electrometer, temperature, pressure, and humidity, and the above process was conducted under IAEA TRS-398^44–46^. Our physical irradiation measurement system is an internationally recognized calibration and testing laboratory and belongs to the Korea Laboratory Certification System (KOLAS). For the dose–response curve, blood samples from two donors were irradiated at 0.5, 1, 2, 3, and 4 Gy; for the blind test, blood from the other two donors was irradiated at 0.5, 2, and 4 Gy, and 1 and 3 Gy, respectively. After 2 h of incubation at 37 °C in a water bath, peripheral blood mononuclear cells (PBMCs) were isolated from the blood using BD Vacutainer CPT tubes (BD Biosciences). The cells were collected using pre-cold RPMI 1640 medium (Welgene, Daegu, Republic of Korea) to prevent cell aggregation. Thereafter, the cells were cultured in pre-warmed RPMI 1640 medium supplemented with 20% FBS (HyClone Laboratories Inc., USA) and 1% kanamycin (Gibco, Canada). To induce metaphase, 2% phytohemagglutinin (PHA; Invitrogen, Gaithersburg, MD, USA) and colcemid (final concentration, 100 ng/mL; Life Technologies, Canada) were simultaneously added to the culture media and incubated at 37 °C in a humidified 5% CO_2_ atmosphere for 48 h. The cells were harvested and treated with pre-warmed 0.075 M hypotonic potassium chloride solution and fixed with methanol/acetic acid (3:1) according to the IAEA guideline^17^. The fixed cells were stained with 5% Giemsa solution for 10 min on a microscope slide (Matsunami, Osaka, Japan). The Metafer4 System (MetaSystems Hard & Software GmbH, Altlussheim, Germany) auto-captured metaphases at 63 × magnification, the images were exported as a JPEG file, and the DLADES identified the dicentric and monocentric chromosomes.

### Data sorting

A two-step data sorting was performed to construct a dose–response curve and calculate a blind-test estimated dose without human review. First, images classified as “Accepted” through the DLADES were sorted. Second, the outlier range was set using an interquartile range (IQR)^47^. The IQR was derived by dividing the number of chromosomes into quarters and calculating the difference between the highest (Q3) and lowest (Q1) quartiles. Next, outliers below Q1-1.5IQR or above Q3 + 1.5IQR were excluded.

The dispersion index (DI) and *u*-test were used to determine the Poisson distribution of dicentric chromosomes per metaphase cell. The DI was calculated as the ratio of the number of dicentric chromosome variances to the frequency with Eq. (1):

1 $${\mathrm{DI}}=\sigma^{2}/y$$

The null hypothesis, where DI = 1, was tested using Papworth’s u-test^48^ at a significance level of 0.05 (|u value|> 1.96); *u*-values > 1.96 indicate overdispersion, whereas values < −1.96 indicate underdispersion.

### Dose–response curve fitting with R

The dose–response curve was fitted with R 3.6.1(R Core Team) using the generalized linear regression models of the R-script provided by Braselmann^17^. The upper and lower 95% confidence intervals for the yield uncertainty were calculated and derived according to Eq. (2):

2 $$Y=C+\alpha D+\upbeta {D}^{2}\pm R\sqrt{\mathrm{var}C+\mathrm{var}\alpha {D}^{2}+\mathrm{var}\beta {D}^{4}+2\mathrm{covar}\left(C,\alpha \right)D+2\mathrm{covar}\left(C,\beta \right){D}^{2}+2\mathrm{covar}(\alpha ,\beta ){D}^{3}}$$

where, *Y* is the dicentric chromosome yield per dose and *D* is the radiation dose (Gy). The regression confidence factor R^2^ was used as the 95% confidence limit of the chi-squared distribution with three degrees of freedom (7.81) for the upper and lower linear-quadratic curves^17,20^. Covariance was calculated using the vcov() function in R 3.6.1.

### Dose estimation with R

Merkle’s approach was used for estimating dose of the blind test sample^17^. Briefly, the upper and lower 95% confidence limits of the number of dicentric chromosomes in the blind sample and the lower and upper yields were calculated using the Poisson test () function in R 3.6.1. The estimated dose was obtained using Eq. (3):

3 $$D=\frac{-\alpha +\sqrt{{\alpha }^{2}+4\beta (Y-C)}}{2\beta }$$

For the upper confidence limit of the estimated dose, *D* is calculated by including the upper yield into *Y* according to Eq. (3) using the lower curve coefficient. For the lower confidence limit of the estimated dose, *D* is calculated by including the lower yield into *Y* according to Eq. (3) using the upper curve coefficient.

### Ethics approval and consent to participate

The study was conducted in accordance with the tenets of the Declaration of Helsinki, and the protocol was approved by the Institutional Review Board of the Dongnam Institute of Radiological and Medical Sciences (DIRAMS) for all experimental procedures (Approval No. D-1602-002-001). Prior to inclusion in the study, informed consent was obtained from all participants under the supervision of the IRB of the DIRAMS via signing of a consent form containing the necessary details about the study. All methods were performed in accordance with the relevant guidelines and regulations.

## Supplementary Information

Supplementary Information.

## Data Availability

The authors confirm that the data supporting the findings of this study are available within the article and its supplementary materials.
