# Supplementary material for: Dicentric chromosome assay using a deep learning-based automated system
Source: Sci Rep. 2022 Dec 21;12:22097. doi: 10.1038/s41598-022-25856-1 (PMC9772420; doi:10.1038/s41598-022-25856-1)
Supplement: Supplementary file 1 — Supplementary Information. [file 41598_2022_25856_MOESM1_ESM.docx]

**Supplementary material**

**Supplementary Table S1.** Automatically identified dicentric chromosome number in Total and Accepted data.

|  |  | Dose (Gy) | Scored  cells | Dic | Dic/cell | Distribution of dicentrics | | | | | | | | DI | u-test | Overdispersion |
| --- | --- | --- | --- | --- | --- | --- | --- | --- | --- | --- | --- | --- | --- | --- | --- | --- |
|  |  |  |  |  |  |  |  |  |  |  |  |  |  |  |  |  |
|  |  |  |  |  |  | 0 | 1 | 2 | 3 | 4 | 5 | 6 | 7 |  |  |  |
| Total | S1 | 0 | 5528 | 453 | 0.08 | 5104 | 395 | 29 | 0 | 0 | 0 | 0 | 0 | 1.05 | 2.44 | Yes |
|  |  | 0.5 | 6139 | 769 | 0.13 | 5439 | 633 | 65 | 2 | 0 | 0 | 0 | 0 | 1.06 | 3.30 | Yes |
|  |  | 1 | 5643 | 941 | 0.17 | 4803 | 749 | 81 | 10 | 0 | 0 | 0 | 0 | 1.07 | 3.69 | Yes |
|  |  | 2 | 6011 | 1757 | 0.29 | 4546 | 1214 | 216 | 30 | 4 | 1 | 0 | 0 | 1.09 | 5.20 | Yes |
|  |  | 3 | 5755 | 2673 | 0.46 | 3742 | 1480 | 429 | 87 | 12 | 4 | 1 | 0 | 1.15 | 7.89 | Yes |
|  |  | 4 | 3669 | 2383 | 0.65 | 2026 | 1081 | 422 | 113 | 19 | 5 | 3 | 0 | 1.16 | 7.06 | Yes |
|  | S2 | 0 | 5100 | 263 | 0.05 | 4849 | 240 | 10 | 1 | 0 | 0 | 0 | 0 | 1.05 | 2.40 | Yes |
|  |  | 0.5 | 5200 | 458 | 0.09 | 4767 | 410 | 21 | 2 | 0 | 0 | 0 | 0 | 1.03 | 1.53 |  |
|  |  | 1 | 6000 | 817 | 0.14 | 5249 | 693 | 50 | 8 | 0 | 0 | 0 | 0 | 1.05 | 2.47 | Yes |
|  |  | 2 | 6000 | 1920 | 0.32 | 4381 | 1362 | 219 | 33 | 4 | 1 | 0 | 0 | 1.05 | 2.57 | Yes |
|  |  | 3 | 6800 | 3628 | 0.53 | 4121 | 1924 | 597 | 127 | 26 | 5 | 0 | 0 | 1.12 | 6.96 | Yes |
|  |  | 4 | 2000 | 1501 | 0.75 | 971 | 676 | 271 | 56 | 18 | 6 | 1 | 1 | 1.11 | 3.38 | Yes |
| Accepted | S1 | 0 | 3524 | 251 | 0.07 | 3286 | 225 | 13 | 0 | 0 | 0 | 0 | 0 | 1.03 | 1.37 |  |
|  |  | 0.5 | 4027 | 468 | 0.12 | 3593 | 401 | 32 | 1 | 0 | 0 | 0 | 0 | 1.03 | 1.51 |  |
|  |  | 1 | 3522 | 506 | 0.14 | 3056 | 428 | 36 | 2 | 0 | 0 | 0 | 0 | 1.02 | 0.95 |  |
|  |  | 2 | 4315 | 1274 | 0.30 | 3233 | 909 | 156 | 15 | 2 | 0 | 0 | 0 | 1.04 | 1.83 |  |
|  |  | 3 | 3915 | 1732 | 0.44 | 2563 | 1030 | 272 | 42 | 8 | 0 | 0 | 0 | 1.07 | 3.23 | Yes |
|  |  | 4 | 2409 | 1621 | 0.67 | 1268 | 759 | 296 | 75 | 10 | 1 | 0 | 0 | 1.06 | 1.97 | Yes |
|  | S2 | 0 | 3382 | 150 | 0.04 | 3237 | 140 | 5 | 0 | 0 | 0 | 0 | 0 | 1.02 | 0.93 |  |
|  |  | 0.5 | 2992 | 223 | 0.07 | 2778 | 205 | 9 | 0 | 0 | 0 | 0 | 0 | 1.01 | 0.25 |  |
|  |  | 1 | 4112 | 504 | 0.12 | 3641 | 440 | 29 | 2 | 0 | 0 | 0 | 0 | 1.02 | 0.75 |  |
|  |  | 2 | 4176 | 1252 | 0.30 | 3096 | 927 | 134 | 19 | 0 | 0 | 0 | 0 | 1.01 | 0.25 |  |
|  |  | 3 | 4267 | 2071 | 0.49 | 2655 | 1220 | 332 | 53 | 7 | 0 | 0 | 0 | 1.03 | 1.37 |  |
|  |  | 4 | 1321 | 880 | 0.67 | 676 | 462 | 146 | 25 | 9 | 3 | 0 | 0 | 1.03 | 0.71 |  |

Dic: Dicentric chromosome, DI: Dispersion Index.

**Supplementary Table S2.** Automatically identified dicentric chromosome number in pooled Accepted data.

|  |  | Dose (Gy) | Scored  cells | Dic | Dic/cell | Distribution of dicentrics | | | | | | | | DI | u-test | Overdispersion |
| --- | --- | --- | --- | --- | --- | --- | --- | --- | --- | --- | --- | --- | --- | --- | --- | --- |
|  |  |  |  |  |  |  |  |  |  |  |  |  |  |  |  |  |
|  |  |  |  |  |  | 0 | 1 | 2 | 3 | 4 | 5 | 6 | 7 |  |  |  |
| Accepted data set pooled | | 0 | 6906 | 401 | 0.06 | 6523 | 365 | 18 | 0 | 0 | 0 | 0 | 0 | 1.03 | 1.87 |  |
|  |  | 0.5 | 7019 | 691 | 0.10 | 6371 | 606 | 41 | 1 | 0 | 0 | 0 | 0 | 1.03 | 1.72 |  |
|  |  | 1 | 7634 | 1010 | 0.13 | 6697 | 868 | 65 | 4 | 0 | 0 | 0 | 0 | 1.02 | 1.26 |  |
|  |  | 2 | 8491 | 2526 | 0.30 | 6329 | 1836 | 290 | 34 | 2 | 0 | 0 | 0 | 1.02 | 1.47 |  |
|  |  | 3 | 8182 | 3803 | 0.46 | 5218 | 2250 | 604 | 95 | 15 | 0 | 0 | 0 | 1.05 | 3.21 | Yes |
|  |  | 4 | 3730 | 2501 | 0.67 | 1944 | 1221 | 442 | 100 | 19 | 4 | 0 | 0 | 1.05 | 2.00 | Yes |

Dic: Dicentric chromosome, DI: Dispersion Index.

**Supplementary Table S3.** Automatically identified dicentric chromosome number in outlier removed data.

|  | Dose (Gy) | Scored  cells | Dic | Dic/cell | Distribution of dicentrics | | | | | | | | DI | | u-test | | Overdispersion | |  |
| --- | --- | --- | --- | --- | --- | --- | --- | --- | --- | --- | --- | --- | --- | --- | --- | --- | --- | --- | --- |
|  |  |  |  |  |  |  |  |  |  |  |  |  |  |  |  |  |  |  |  |
|  |  |  |  |  | 0 | 1 | 2 | 3 | 4 | 5 | 6 | 7 | |  | |  | |  | |
| S1 | 0 | 3281 | 245 | 0.07 | 3049 | 219 | 13 | 0 | 0 | 0 | 0 | 0 | | 1.03 | | 1.29 | |  | |
|  | 0.5 | 3497 | 445 | 0.13 | 3086 | 378 | 32 | 1 | 0 | 0 | 0 | 0 | | 1.03 | | 1.27 | |  | |
|  | 1 | 3196 | 480 | 0.15 | 2755 | 404 | 35 | 2 | 0 | 0 | 0 | 0 | | 1.02 | | 0.84 | |  | |
|  | 2 | 3848 | 1211 | 0.31 | 2825 | 854 | 152 | 15 | 2 | 0 | 0 | 0 | | 1.03 | | 1.35 | |  | |
|  | 3 | 3795 | 1711 | 0.45 | 2461 | 1015 | 269 | 42 | 8 | 0 | 0 | 0 | | 1.07 | | 2.93 | | Yes | |
|  | 4 | 2193 | 1551 | 0.71 | 1109 | 712 | 289 | 72 | 10 | 1 | 0 | 0 | | 1.03 | | 1.15 | |  | |
| S2 | 0 | 3381 | 149 | 0.04 | 3237 | 139 | 5 | 0 | 0 | 0 | 0 | 0 | | 1.02 | | 0.96 | |  | |
|  | 0.5 | 2813 | 216 | 0.08 | 2606 | 198 | 9 | 0 | 0 | 0 | 0 | 0 | | 1.01 | | 0.26 | |  | |
|  | 1 | 3626 | 466 | 0.13 | 3189 | 410 | 25 | 2 | 0 | 0 | 0 | 0 | | 1.00 | | 0.20 | |  | |
|  | 2 | 3943 | 1216 | 0.31 | 2895 | 899 | 130 | 19 | 0 | 0 | 0 | 0 | | 1.00 | | -0.03 | |  | |
|  | 3 | 3884 | 1966 | 0.51 | 2359 | 1149 | 318 | 51 | 7 | 0 | 0 | 0 | | 1.02 | | 0.70 | |  | |
|  | 4 | 1210 | 839 | 0.69 | 598 | 436 | 140 | 24 | 9 | 3 | 0 | 0 | | 1.01 | | 0.32 | |  | |
| pooled | 0 | 6662 | 394 | 0.06 | 6286 | 358 | 18 | 0 | 0 | 0 | 0 | 0 | | 1.03 | | 1.87 | |  | |
|  | 0.5 | 6310 | 661 | 0.10 | 5692 | 576 | 41 | 1 | 0 | 0 | 0 | 0 | | 1.03 | | 1.60 | |  | |
|  | 1 | 6822 | 946 | 0.14 | 5944 | 814 | 60 | 4 | 0 | 0 | 0 | 0 | | 1.01 | | 0.80 | |  | |
|  | 2 | 7791 | 2427 | 0.31 | 5720 | 1753 | 282 | 34 | 2 | 0 | 0 | 0 | | 1.01 | | 0.93 | |  | |
|  | 3 | 7679 | 3677 | 0.48 | 4820 | 2164 | 587 | 93 | 15 | 0 | 0 | 0 | | 1.04 | | 2.56 | | Yes | |
|  | 4 | 3403 | 2390 | 0.70 | 1707 | 1148 | 429 | 96 | 19 | 4 | 0 | 0 | | 1.03 | | 1.11 | |  | |

Dic: Dicentric chromosome, DI: Dispersion Index.

**Supplementary Table S4.** Number of automatically identified dicentric chromosomes for the blind test.

|  |  | Dose (Gy) | Scored  cells | Dic | Dic/cell | Distribution of dicentrics | | | | | | | | DI | u-test | Overdispersion |
| --- | --- | --- | --- | --- | --- | --- | --- | --- | --- | --- | --- | --- | --- | --- | --- | --- |
|  |  |  |  |  |  |  |  |  |  |  |  |  |  |  |  |  |
|  |  |  |  |  |  | 0 | 1 | 2 | 3 | 4 | 5 | 6 | 7 |  |  |  |
| Total | Blind test 1 | 0.5 | 7258 | 719 | 0.10 | 6598 | 604 | 53 | 3 | 0 | 0 | 0 | 0 | 1.07 | 4.43 | Yes |
|  |  | 1 | 7134 | 903 | 0.13 | 6302 | 766 | 61 | 5 | 0 | 0 | 0 | 0 | 1.04 | 2.50 | Yes |
|  |  | 3 | 7050 | 2654 | 0.38 | 4943 | 1650 | 377 | 70 | 10 | 0 | 0 | 0 | 1.11 | 6.61 | Yes |
|  | Blind test 2 | 2 | 6215 | 1767 | 0.28 | 4720 | 1255 | 212 | 24 | 4 | 0 | 0 | 0 | 1.06 | 3.59 | Yes |
|  |  | 4 | 2191 | 1643 | 0.75 | 1100 | 683 | 293 | 90 | 21 | 4 | 0 | 0 | 1.14 | 4.57 | Yes |
| Accepted | Blind test 1 | 0.5 | 3722 | 320 | 0.09 | 3431 | 263 | 27 | 1 | 0 | 0 | 0 | 0 | 1.10 | 4.40 | Yes |
|  |  | 1 | 4231 | 597 | 0.14 | 3680 | 510 | 36 | 5 | 0 | 0 | 0 | 0 | 1.03 | 1.38 |  |
|  |  | 3 | 4086 | 1859 | 0.45 | 2603 | 1166 | 265 | 45 | 7 | 0 | 0 | 0 | 1.02 | 0.94 |  |
|  | Blind test 2 | 2 | 4345 | 1237 | 0.28 | 3270 | 931 | 127 | 16 | 1 | 0 | 0 | 0 | 1.01 | 0.38 |  |
|  |  | 4 | 1617 | 1259 | 0.78 | 746 | 568 | 230 | 61 | 12 | 0 | 0 | 0 | 0.99 | -0.21 |  |
| Outlier removed data | Blind test 1 | 0.5 | 3190 | 292 | 0.09 | 2924 | 240 | 26 | 0 | 0 | 0 | 0 | 0 | 1.09 | 3.48 | Yes |
|  |  | 1 | 3795 | 574 | 0.15 | 3267 | 487 | 36 | 5 | 0 | 0 | 0 | 0 | 1.03 | 1.16 |  |
|  |  | 3 | 3563 | 1719 | 0.48 | 2196 | 1069 | 250 | 42 | 6 | 0 | 0 | 0 | 1.00 | -0.12 |  |
|  | Blind test 2 | 2 | 3847 | 1188 | 0.31 | 2820 | 884 | 126 | 16 | 1 | 0 | 0 | 0 | 0.99 | -0.24 |  |
|  |  | 4 | 1552 | 1239 | 0.80 | 696 | 557 | 227 | 60 | 12 | 0 | 0 | 0 | 0.98 | -0.68 |  |

Dic: Dicentric chromosome, DI: Dispersion Index.
